# Proposed new clinicopathological surrogate definitions of luminal A and luminal B (HER2-negative) intrinsic breast cancer subtypes

**DOI:** 10.1186/bcr3679

**Published:** 2014-06-20

**Authors:** Patrick Maisonneuve, Davide Disalvatore, Nicole Rotmensz, Giuseppe Curigliano, Marco Colleoni, Silvia Dellapasqua, Giancarlo Pruneri, Mauro G Mastropasqua, Alberto Luini, Fabio Bassi, Gianmatteo Pagani, Giuseppe Viale, Aron Goldhirsch

**Affiliations:** 1 Division of Epidemiology and Biostatistics, European Institute of Oncology, Via Ripamonti 435, Milan 20141, Italy; 2 Division of Early Drug Development for Innovative Therapies, European Institute of Oncology, Via Ripamonti 435, Milan 20141, Italy; 3 Division of Medical Senology, Department of Medicine, European Institute of Oncology, Via Ripamonti 435, Milan 20141, Italy; 4 Department of Pathology and Laboratory Medicine, European Institute of Oncology, Via Ripamonti 435, Milan 20141, Italy; 5 Division of Breast Surgery, European Institute of Oncology, Via Ripamonti 435, Milan 20141, Italy; 6 School of Medicine, University of Milan, Via Ripamonti 435, Milan 20141, Italy; 7 Breast Cancer Program, European Institute of Oncology, Via Ripamonti 435, Milan 20141, Italy

## Abstract

**Introduction:**

The St Gallen International Expert Consensus on the Primary Therapy of Early Breast Cancer 2013 recognized substantial progress in the pathological characterization of breast cancer subtypes. A useful surrogate definition was developed to distinguish luminal A–like breast cancer from luminal B–like disease based on a combination of estrogen receptor (ER), progesterone receptor (PgR) and Ki-67 status, without a requirement for molecular diagnostics. Differences depend upon the choice of the threshold value for Ki-67 and the requirement for substantial PgR positivity. We aimed to verify the suitability of the new surrogate definitions of luminal subtypes in terms of distant disease control in a large series of patients.

**Methods:**

We studied 9,415 women with a median follow-up of 8.1 years who (1) had ER-positive, human epidermal growth factor receptor 2 (HER2)–negative early breast cancer and (2) had undergone surgery at the European Institute of Oncology between 1994 and 2006. We evaluated distant disease-free survival of patients with “low” (<14%), “intermediate” (14% to 19%) or “high” (≥20%) Ki-67 positivity stratified by PgR expression (negative or low versus high). We calculated the cumulative incidence of distant events, considered competing events and performed multivariable analysis adjusted for pathologic tumor stage, pathologic node stage, tumor grade, peritumoral vascular invasion and menopausal status.

**Results:**

Lack of substantial PgR positivity was associated with poorer outcomes only for patients with an intermediate Ki-67 level (*P* < 0.001). The 4,890 patients (51.9%) with low Ki-67 level (any PgR expression level) or with intermediate Ki-67 level but substantial PgR positivity had comparably good outcomes and thus may represent a most advantageous grouping of those with luminal A–like disease.

**Conclusions:**

The updated pathological definition of intrinsic molecular subtypes may maximize the number of patients classified as having the luminal A–like intrinsic subtype of breast cancer and for whom the use of cytotoxic drugs could mostly be avoided.

## Introduction

At the 13th St Gallen International Breast Cancer Conference in 2013, an international expert panel reviewed the clinicopathological surrogate definitions of breast cancer subtypes [1]. On the basis particularly of results of the Prat *et al*. study [2], the panel advised that the clarity of the distinction between “luminal A–like” and “luminal B–like” tumors could be improved from the previous consensus guidelines [3] by including a requirement for substantial progesterone receptor (PgR) positivity (≥20%) in the definition of “luminal A–like” disease. As described in the expert consensus report, adding this restriction will have the effect of reducing the number of patients classified as having luminal A–like cancer and thus increase the number for whom cytotoxic therapy is generally recommended. Concurrently, the expert panel removed the threshold value for “high Ki-67” in the surrogate definition of the luminal B subtype. A level of Ki-67 ≥ 14% was previously proposed for the distinction of luminal A and luminal B intrinsic subtypes, but, on the basis of a comparison in which gene array data were utilized [4], a majority of the panel voted that a threshold of ≥20% is indicative of high Ki-67 status [1]. Although not explicitly recommended, application of this new cut point will have the opposite effect of increasing the number of patients classified as having luminal A–like breast cancer and thus decreasing the number for whom cytotoxic therapy is generally recommended.

In order to verify the suitability of the new surrogate definitions of intrinsic breast cancer subtypes, we retrieved information on a large series of women treated for a first primary nonmetastatic breast cancer at the European Institute of Oncology (IEO) (Milan, Italy) between 1994 and 2006 and compared their outcomes according to the surrogate definitions of intrinsic subtypes proposed at the 12th St Gallen International Breast Cancer Conference and also according to the revised definition proposed at the 13th conference [1].

## Methods

### Study population

The initial data set used for this analysis comprised all women who had undergone surgery for a first primary breast cancer at the IEO between 1994 and 2006 and had not received neoadjuvant treatment. Data were prospectively collected from the institutional breast cancer database. Women with metastatic breast disease at the time of presentation or established within 3 months after surgery were excluded. For 1,207 (9.1%) of the 13,265 remaining patients, assessment of human epidermal growth factor receptor 2 (HER2) status was missing, thus impeding the determination of intrinsic molecular tumor subtypes. For the purposes of the study, an additional 1,669 patients (12.6%) with HER2-positive tumors (1,008 endocrine-responsive and 661 endocrine-nonresponsive) and 974 patients (7.3%) with triple-negative tumors were also excluded, because no modifications were made in the revised consensus for the definitions of the “luminal B (HER2-positive)”, “HER2-positive” or “triple-negative” breast cancer subtypes. The final data set comprises 9,415 patients with endocrine-responsive, HER2-negative breast cancer.

Our use of the data was approved by the ethics committee of the IEO and by the Italian Data Protection Authority (Garante per la protezione dei dati personali), and the need for individual patients’ consent was waived.

### Laboratory methods

Tumors were classified histologically according to the World Health Organization’s histological classification system as modified by Rosen and Obermann [5]. Tumor grade was evaluated according to the classification scheme described by Elston and Ellis [6]. Immunohistochemical staining for estrogen receptor (ER), PgR, HER2 protein and Ki-67 antigen was performed with consecutive tissue sections from the same tumor blocks. The following primary antibodies were used: the monoclonal antibody (mAb) against ER (clone 1D5, 1:100 dilution; Dako, Glostrup, Denmark), the mAb against PgR (clone 1A6, 1:800 dilution; Dako), the MIB-1 mAb against Ki-67 antigen (1:100 dilution; Dako) and the polyclonal antibody against HER2 protein (1:3,200 dilution; Dako). Only nuclear reactivity was taken into account for ER, PgR and Ki-67, irrespective of the staining intensity, whereas only intense and complete membrane staining in >10% of the tumor cells were considered HER2 overexpression (3+). In addition, fluorescence *in situ* hybridization assays were performed for the final determination of HER2 status for tumors with 2+ immunoreactivity.

### Follow-up and outcomes

Patients were followed for recurrence or death according to clinical protocols. For those no longer attending clinical visits at our institute, essential follow-up information was collected by telephone. For those lost to follow-up, information on vital status was obtained from the municipal vital statistics office. For the purposes of this report, the main outcome was distant disease-free survival (DDFS), taking into consideration development of metastasis in distant organs and death due to breast cancer as primary events.

### Statistical analysis

Associations between categorical and ordinal variables and PgR or Ki-67 status were evaluated with the χ^2^ test. DDFS duration was calculated from the date of surgery until the date of any first event or the date of last contact with the patient. Curves of the cumulative incidence of events were drawn considering metastases and deaths due to breast cancer as events and locoregional events, contralateral tumors, nonbreast primary tumors and deaths due to other or unknown causes considered as competing events. Cumulative incidences were compared across different subgroups by means of the Gray test. Multivariable Cox proportional hazards models were used to evaluate risk of metastasis or death due to breast cancer across groups, adjusting for pathologic tumor stage (pT), pathologic node stage (pN), tumor grade, peritumoral vascular invasion and menopausal status. All analyses were carried out with SAS software (version 9.3; SAS Institute, Cary, NC, USA). All *P*-values are two-sided.

## Results

**Figure 1:**
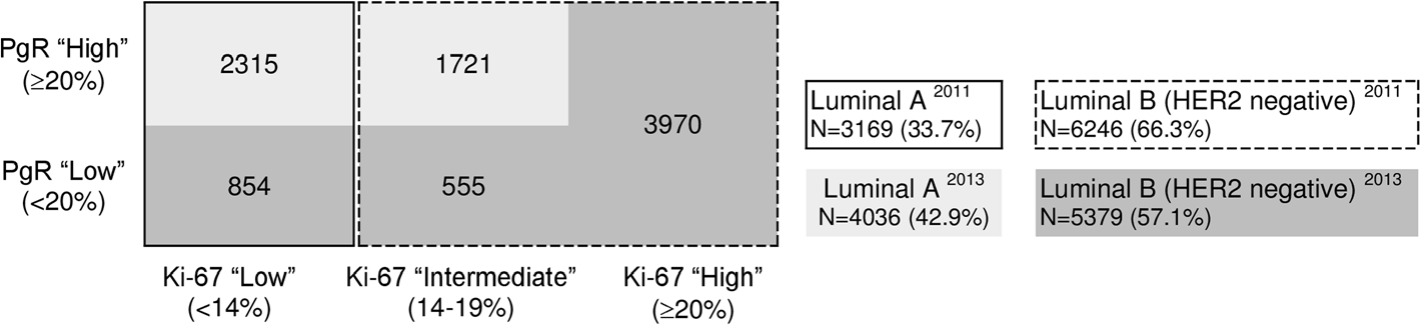
**Patient distribution according to molecular characteristics and St Gallen 2011 and 2013 intrinsic molecular subtype definitions.** The threshold of ≥14% for Ki-67 in the 2011 definition was derived from comparison with gene array data as a prognostic factor [3,4], whereas the threshold of ≥20% in the 2013 definition was approved by the majority of the expert consensus panel [1]. HER2, Human epidermal growth factor receptor 2; PgR, Progesterone receptor.

**Figure 2:**
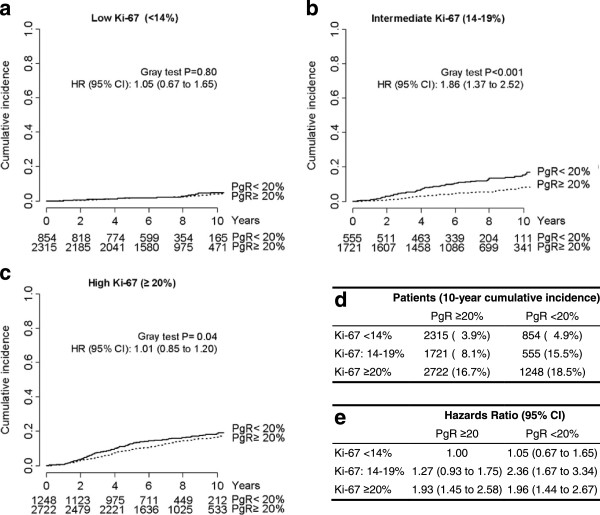
**Distant disease-free survival according to Ki-67 and progesterone receptor expression levels. (a)** Patients with low Ki-67 levels (<14%). **(b)** Patients with intermediate Ki-67 expression (14% to 19%). **(c)** Patients with high Ki-67 expression (≥20%). **(d)** Number of patients and 10-year cumulative incidence. **(e)** Multivariable analysis. PgR, Progesterone receptor. Hazard ratios (HRs) and 95% confidence intervals (CIs) were calculated using a multivariable Cox proportional hazards regression model adjusted for pathologic tumor stage, pathologic node stage, tumor grade, peritumoral vascular invasion and menopausal status.

The characteristics of the 9,415 patients who had surgery for endocrine-responsive, HER2-negative breast cancer and who satisfied the eligibility criteria are described in Table 1 and Additional file 1: Table S1. Among those patients, 4,278 (45.4%) were in premenopause. The tumors were ductal in 7,114 cases (75.6%), lobular in 1,150 cases (12.2%), mixed ductal and lobular in 397 cases (4.2%) and of other histological subtype in the remaining 754 cases (8.0%). A total of 6,601 (70.1%) of the patients had pT1 disease, and 5,510 patients (58.5%) had no regional lymph node involvement (pNx/or pN0). A total of 7,777 (82.6%) of the women had undergone breast-conserving surgery, 7,999 (85.0%) had received adjuvant radiotherapy and 3,029 (32.2%) had been treated with adjuvant chemotherapy. During a median follow-up of 8.1 years (IQR = 6.1 to 10.2 years), 592 women (6.3%) had a locoregional relapse, 852 (9.0%) developed a distant metastasis and 822 (8.7%) had some other event (contralateral breast cancer, other primary or death) (Table 1).Totals of 3,169 (33.7%) of the patients had luminal A–like breast cancer and 6,246 (66.3%) had luminal B–like breast cancer on the basis of the initial surrogate definition of intrinsic molecular subtypes proposed by the international expert consensus panel in 2011 (Figure 1). When we applied the 2013 updated St Gallen definition of intrinsic subtypes, we found that 854 (26.9%) of the 3,169 tumors previously classified as luminal A–like were now luminal B–like tumors because they were associated with negative or low PgR levels. Conversely, 1,721 (75.6%) of the 2,276 initially luminal B–like tumors associated with intermediate Ki-67 levels (that is, between 14% and 20%) had PgR expression levels ≥20% and thus were reclassified as luminal A–like tumors. As a result, 4,036 (42.9%) of the patients were reclassified as having luminal A–like disease and 5,379 (57.1%) were considered to have luminal B–like breast cancer (Figure 1).In order to verify whether the single modifications improved the categorization of patients in terms of outcomes, we evaluated the long-term DDFS of patients with low (<14%), intermediate (14% to 19%) and high (≥20%) Ki-67 levels, stratified according to PgR level (<20%vs. ≥20%) (Figure 2).

**Table 1:** **Characteristics of the patients**^**a**^

**Variables**	**Number of patients (%)**
All	9,415 (100)
Age at surgery, yr	
<35	309 (3.3)
35 to 50	3,809 (40.5)
51 to 65	3,637 (38.6)
>65	1,660 (17.6)
Menopausal status	
Premenopausal	4,278 (45.4)
Postmenopausal	5,137 (54.6)
Histological tumor subtype	
Ductal	7,114 (75.6)
Lobular	1,150 (12.2)
Mixed	397 (4.2)
Other	754 (8.0)
pT	
pT1	6,601 (70.1)
pT2	2,514 (26.7)
pT3/4	300 (3.2)
pN	
pNx	309 (3.3)
pN0	5,201 (55.2)
pN+	3,905 (41.5)
Tumor grade	
G1	2,160 (22.9)
G2	4,930 (52.4)
G3	2,007 (21.3)
NA	318 (3.4)
ER positivity	
≥20%	9,228 (98.0)
<20%	187 (2.0)
PgR positivity	
≥20%	6,758 (71.8)
<20%	2,657 (28.2)
Ki-67 level	
<14%	3,169 (33.7)
14% to 19%	2,276 (24.2)
≥20%	3,970 (42.2)
PVI	
Absent	7,039 (74.8)
Present	2,376 (25.2)
Surgery	
Quadrantectomy	7,777 (82.6)
Mastectomy	1,638 (17.4)
Radiotherapy	
No	1,416 (15.0)
Yes	7,999 (85.0)
Chemotherapy	
No	6,386 (67.8)
Yes	3,029 (32.2)
Competing risk^b^	
No events	7,149 (75.9)
Locoregional relapse	592 (6.3)
Distant metastasis	852 (9.0)
Other	822 (8.7)

^a^ER, Estrogen receptor; NA, Not applicable; PgR, Progesterone receptor; pN, Pathologic node stage; pT, Pathologic tumor stage; PVI, Peritumoral vascular invasion. ^b^During a median follow-up time, estimated in nonevents, of 8.1 years (IQR = 6.1 to 10.2 years).

The DDFS durations of the 854 women with low Ki-67 expression and low PgR levels (that is, those who were reclassified as having luminal B–like instead of luminal A–like breast cancer according to the 2013 definitions) were similar to those of the 2,315 women with low Ki-67 expression and high PgR levels (that is, those who remained classified as having luminal A–like breast cancer) (hazard ratio (HR) = 1.05, 95% confidence interval (CI) = 0.67 to 1.65; *P* = 0.80 by Gray test) (Figure 2a).

The DDFS durations of the 1,721 women with intermediate Ki-67 expression and high PgR levels (that is, those who were reclassified as having luminal A–like instead of luminal B–like breast cancer according to the 2013 definitions) were significantly better than those of the remaining 555 women with intermediate Ki-67 expression and low PgR levels (HR = 1.86, 95% CI = 1.37 to 2.52; *P* < 0.001 by Gray test) (Figure 2b).

Among women with high Ki-67 expression levels (that is, those classified as having luminal B–like in both sets of definitions), the DDFS durations of the 2,722 women with high PgR levels were significantly better than those of the remaining 1,248 women with low PgR levels as assessed in univariate analysis (*P* = 0.04 by Gray test), but the association disappeared in the multivariable analysis (HR = 1.01, 95% CI = 0.85 to 1.20, P = 0.88) (Figure 2c).The cumulative incidence of distant metastasis (or breast cancer–related death as a first event) was evenly low for women with low Ki-67 expression and high PgR levels (3.9%), for women with low Ki-67 expression and low PgR levels (4.9%) and for women with intermediate Ki-67 expression and high PgR levels (8.1%). In contrast, it was evenly high for women with intermediate Ki-67 expression and low PgR levels (15.5%), for women with high Ki-67 expression and high PgR levels (16.7%) and for women with high Ki-67 expression and low PgR levels (18.5%) (Figure 2d).Multivariate analysis confirmed statistically significant increased risks of distant metastasis for women with intermediate Ki-67 expression and low PgR levels (HR = 2.36, 95% CI = 1.67 to 3.34, P < 0.0001), for women with high Ki-67 expression and high PgR levels (HR = 1.93, 95% CI = 1.45 to 2.58, P < 0.0001) and for women with high Ki-67 expression and low PgR levels (HR = 1.96, 95% CI = 1.44 to 2.67, P < 0.0001) compared to women with low Ki-67 expression and high PgR levels. No significant increased risk of distant metastasis was observed for women with low Ki-67 expression and low PgR levels (HR = 1.05, 95% CI = 0.67 to 1.65, P = 0.81) and for women with intermediate Ki-67 expression and high PgR levels (HR = 1.27, 95% CI = 0.93 to 1.75, P = 0.14), again, compared to women with low Ki-67 expression and high PgR levels (Figure 2e).

### Proposal for new definitions of intrinsic molecular subtypes

On the basis of our findings, we propose new definitions of the luminal intrinsic molecular subtypes which would better stratify patients in terms of DDFS. We would classify the two subtypes as follows. (1) Luminal A–like tumors are ER-positive and HER2-negative with low Ki-67 expression (<14%) or with intermediate Ki-67 expression (14% to 19%) and high PgR levels (≥20%). (2) Luminal B–like (HER2-negative) tumors are ER-positive and HER2-negative with intermediate Ki-67 expression (14% to 19%) and low PgR levels (<20%) or with high Ki-67 expression (≥20%) (Table 2).

**Table 2:** **New proposal for surrogate definitions of intrinsic subtypes of HER2-negative, endocrine-responsive breast cancer**^**a**^

**Intrinsic subtypes**	**Clinicopathological surrogate definitions**
Luminal A	“Luminal A–like”
	*All of*:
	ER-positive
	HER2-negative
	*And at least one of*:
	Ki-67 low expression (<14%)
	Ki-67 intermediate expression (14% to 19%) and PgR high expression (≥20%)
Luminal B (HER2-negative)	“Luminal B–like (HER2-negative)”
	*All of*:
	ER-positive
	HER2-negative
	*And at least one of*:
	Ki-67 intermediate expression (14% to -19) and PgR negative or low expression (<20%)
	Ki-67 high expression (≥20%)

^a^ER, Estrogen receptor; HER2, Human epidermal growth factor receptor 2; PgR, Progesterone receptor.

**Figure 3:**
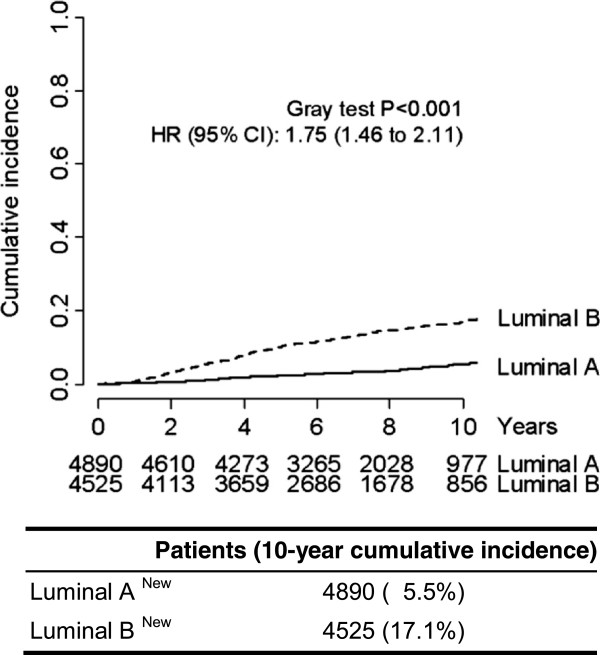
**Distant disease-free survival according to our proposed intrinsic molecular subtypes.** Hazards ratios (HRs) and 95% confidence intervals (CIs) were calculated using a multivariable Cox proportional hazards regression model adjusted for pathologic tumor stage, pathologic node stage, tumor grade, peritumoral vascular invasion and menopausal status.

**Figure 4:**
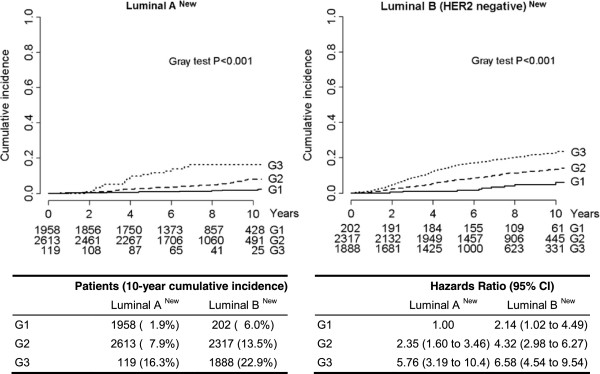
**Distant disease-free survival according to our proposed new definitions of intrinsic molecular subtypes and tumor grades.** Hazard ratios and 95% confidence intervals (CIs) were calculated using a multivariable Cox proportional hazards regression model adjusted for pathologic tumor stage, pathologic node stage, tumor grade, peritumoral vascular invasion and menopausal status. *P* = 0.33 for the interaction between tumor grade and new intrinsic molecular subtype. The percentages of patients with luminal A–like breast cancer who received adjuvant chemotherapy were 9.5% (G1), 24.73% (G2) and 42.0% (G3). Similarly, 21.3%, 38.7% and 60.5% of patients with G1, G2 and G3, luminal B–like breast cancer, respectively, received adjuvant chemotherapy.

As a result, 4,890 women (51.9%) were considered to have luminal A–like and 4,525 (48.1%) to have luminal B–like breast cancer (Additional file 1: Table S3). The cumulative incidence of distant metastasis at 10 years was 5.5% for luminal A–like and 17.1% for luminal B–like breast cancer (Figure 3). After adjustment for potential confounders, the risk (HR) of distant disease for women with luminal B–like versus luminal A–like tumors was 1.75 (95% CI = 1.46 to 2.11, P < 0.0001).According to our proposed new definitions, the majority of luminal A–like tumors in our series were either well-differentiated (41.7%) or moderately differentiated (55.7%), and relatively few were poorly differentiated (2.5%). In contrast, the majority of the luminal B–like tumors were either moderately differentiated (52.6%) or poorly differentiated (42.8%), and relatively few were well-differentiated (4.6%) (Figure 4). The outcomes of the patients with poorly differentiated luminal A–like tumors were not significantly different from those of patients with poorly differentiated luminal B–like tumors. Reciprocally, the outcomes of the few patients with well-differentiated luminal B–like tumors were significantly better than those of patients with moderately or poorly differentiated luminal B–like tumors, but significantly worse than those of women with well-differentiated luminal A–like tumors.

## Discussion

We extrapolated data from a large monoinstitutional series of patients who had undergone surgery for breast cancer to assess the validity and clinical impact, in terms of DDFS, of the modified surrogate updated definitions of the “luminal A” and “luminal B (HER2-negative)” intrinsic molecular subtypes of breast cancer outlined in the 2011 and 2013 versions of the St Gallen International Expert Consensus on the Primary Therapy of Early Breast Cancer guidelines [1,3].

The first change made by the panel was the requirement for substantial PgR positivity (≥20%) in the definition of luminal A–like disease. We found that (1) 854 (26.9%) of the 3,169 women in our series previously classified as having luminal A–like breast cancer (using the threshold value <14% Ki-67 expression) would have been reclassified as having luminal B–like breast cancer and, consequently, (2) most should have been treated with cytotoxic drugs. However, the majority of these 854 patients had well-differentiated or moderately differentiated tumors, did not receive adjuvant chemotherapy and nevertheless experienced similar favorable outcomes compared to patients with tumors with low Ki-67 levels and substantial PgR positivity.

The second modification was the selection, by the majority of the panel experts, of 20% as the threshold value for Ki-67 for the distinction of luminal A and luminal B intrinsic subtypes. We found that 1,721 (75.6%) of the 2,276 patients with tumors with intermediate Ki-67 expression (that is, comprising patients meeting the old and new thresholds) had substantial PgR positivity (≥20%) and had outcomes similar to those of patients with luminal A–like breast cancer, thus confirming the correct classification of this subgroup of patients. We also found that the 555 patients with tumors with intermediate Ki-67 expression but negative or low PgR levels (<20%) had outcomes similar to those of patients with luminal B–like breast cancer. These two observations support the requirement for substantial PgR positivity (≥20%) in the definition of luminal A–like disease among patients with intermediate Ki-67 expression levels (14% to 19%).

Therefore, on the basis of our analysis, we propose new clinicopathological definitions using 20% as a threshold for Ki-67 to distinguish the luminal A and luminal B intrinsic molecular breast cancer subtypes. In addition, we propose a requirement for substantial PgR positivity in the definition of luminal A–like disease only when Ki-67 expression is between 14% and 20% (Table 2). Although we found that luminal A–like tumors represented only 33.7% and 42.9% of all ER-positive, HER2-negative breast cancers when we applied the 2011 and 2013 St Gallen surrogate definitions, respectively, they would represent about 50% of HER2-negative luminal tumors in breast cancer patients according to our proposed new definitions. This proportion is much the same as that observed by study researchers who have defined intrinsic molecular subtypes by utilizing gene expression profiling [7,8]. The major implication of our proposed new distinction between luminal A–like tumors (more endocrine-sensitive, more indolent and better prognosis) and luminal B–like tumors (less endocrine-sensitive, more aggressive and worse prognosis) is the impact on decision-making regarding the utility or futility of adjuvant cytotoxic therapy between these two groups.

Further evidence has accrued in the past several years to support the use of multigene signatures to make distinctions between patients with different luminal disease subtypes. Many different multigene assays provide prognostic information, primarily derived from the sampling of proliferation genes, which emphasizes the need for some measure of proliferation in any surrogate classification [9]. In many settings, however, patients can access multigene testing only by making large personal out-of-pocket payments. Therefore, from a global perspective, multigene testing will remain inaccessible in the immediate future for the majority of women with early breast cancer. It is for these women that the 2013 St Gallen Panel believed that the approach adopted, based on the available clinicopathological testing and now expressed in our proposed immunohistochemistry-based surrogate classification scheme, will be more widely applicable at lesser cost, notwithstanding its limited validation. Our data confirm the clinical validity of our proposed classification definitions. Despite being increased in number by our proposal, only a small proportion of luminal A–like tumors were defined as poorly differentiated (2.5%). Reciprocally, a small proportion of our redefined luminal B–like tumors were well-differentiated (4.6%). Such a pattern was observed in a previous study in which the intrinsic subtype assignment was based on gene expression profiling. Bastien *et al*. reported 14.1% poorly differentiated luminal A tumors and 9.4% well-differentiated luminal B tumors among women with locally advanced primary invasive breast cancer studied using the PAM50 gene set [8]. Interestingly, an accurate assessment of tumor grade remains a powerful prognostic parameter, though it is not included in any of the surrogate definitions for luminal tumors. In our present series, patients with G1 tumors belonging to the luminal B class had significantly better outcomes than patients with G2 and G3 tumors of the same class, despite the fact that almost 80% of the former group of patients did not receive any chemotherapy in addition to endocrine therapy. Conversely, patients with G3 tumors of the luminal A class had worse outcomes than those with G1 and G2 tumors of the same class, with HRs not different from the HR for patients with G3 tumors of the luminal B class, despite the fact that >40% of the former patient group did receive adjuvant chemotherapy. It is tempting to speculate that a more comprehensive surrogate definition of the luminal classes of tumors for clinical purposes could incorporate grade as a first discriminator (G1 for luminal A and G3 for luminal B) and biological features to define the luminal classes among the G2 tumors only. However, the aim of our present study was to verify the suitability of the surrogate definitions of luminal subtypes according to the original parameters proposed by the St Gallen international expert panel.

### Strengths of the study

This study is based on our analysis of a large monoinstitutional series of patients treated for breast cancer in a high-volume reference center where treatment and management are widely standardized. All cases had been discussed individually after surgery by a multidisciplinary team, and all decisions about adjuvant treatment had been made, before the publication of the first St Gallen clinicopathological surrogate definitions. Another important strength is the central pathological examination of all tumor specimens by a dedicated team of highly experienced pathologists, thus guaranteeing maximal reliability and reproducibility of the evaluation of molecular tumor characteristics. This is particularly important because interlaboratory variations in the evaluation of ER, PgR and Ki-67 expression levels can impact the molecular classification of breast cancer when based on fixed threshold values [10-12].

### Study caveats

This is a retrospective study in which patients were treated according to well-defined protocols, but before the dissemination of the definitions set forth in the St Gallen guidelines. Therefore, some patients that we propose to classify as having luminal A–like breast cancer, and for most of whom the use of cytotoxic drugs would not be recommended, might have actually received and benefited from adjuvant chemotherapy. Still, only a small proportion (18.5%) of women with luminal A–like breast cancer received adjuvant chemotherapy, in contrast to almost half (46.9%) of women with luminal B–like breast cancer. We also repeated all of our multivariable analyses, adjusting for the use of adjuvant chemotherapy, and the results remained virtually unchanged (Additional file 1: Table S2).

Another limitation of this study lies in the substantial variability in Ki-67 scoring that has been observed even at some of the world’s most experienced pathological laboratories [11]. In the absence of standardization of the scoring methodology for Ki-67 expression, which is required for using fixed Ki-67 values and cutoffs in clinical decision-making, it is the responsibility of each local laboratory to decide on the Ki-67 thresholds that best apply to their data.

## Conclusions

On the basis of the observed outcomes in a large series of women treated for breast cancer, we propose an updated definition of intrinsic molecular subtypes that may maximize the number of patients classifiable as having luminal A–like breast cancer for whom the use of cytotoxic drugs could largely be avoided. The derived numbers, characteristics and outcomes of patients with the luminal A–like and luminal B–like subtypes under our proposed definitions would be comparable to those observed in studies in which researchers have defined intrinsic molecular subtypes by utilizing gene expression profiling.

## Abbreviations

CI: Confidence interval; DDFS: Distant disease-free survival; ER: Estrogen receptor; HER2: Human epidermal growth factor receptor 2; HR: Hazard ratio; IEO: European Institute of Oncology; PgR: Progesterone receptor; PVI: Peritumoral vascular invasion.

## Competing interests

The authors declare that they have no competing interests.

## Authors' contributions

PM, AG, GV and GC conceived the study. NR, DD, AL, MC, SD, GP, MGM, FB, GP, GP, GV and AG provided study material and assembled the data. DD and PM analyzed the data. DD, PM, GV, AG, GC, MC, MGM and NR interpreted the results. PM drafted the manuscript. All authors contributed to the editing of the final version of the manuscript and approved the final version for publication.

## Supplementary Material

Additional file 1: Table S1 Characteristics of the patients according to Ki-67 and PgR expression levels. **Table S2.** Multivariate analysis for distant disease-free survival. **Table S3.** Characteristics of the patients according to our new proposal for molecular subtype definitions based on outcomes. Click here for file
