# Supplementary material for: Proposed new clinicopathological surrogate definitions of luminal A and luminal B (HER2-negative) intrinsic breast cancer subtypes
Source: Breast Cancer Res. 2014 Jun 20;16(3):R65. doi: 10.1186/bcr3679 (PMC4095689; doi:10.1186/bcr3679)
Supplement: Additional file 1: Table S1 — Characteristics of the patients according to Ki-67 and PgR expression levels. Table S2. Multivariate analysis for distant disease-free survival. Table S3. Characteristics of the patients according to our new proposal for molecular subtype definitions based on outcomes. [file bcr3679-S1.doc]

**Supplementary table 1.** Characteristics of the patients according to Ki-67 and PgR expression levels

| **Variable** | **All** | **Ki-67 <14%** | | | **Ki-67:14-19%** | | | **Ki-67 ≥20%** | | |
| --- | --- | --- | --- | --- | --- | --- | --- | --- | --- | --- |
|  |  | **PgR <20%**  **N (% col)** | **PgR ≥20%**  **N (% col)** | **p** | **PgR <20%**  **N (% col)** | **PgR ≥20%**  **N (% col)** | **p** | **PgR <20%**  **N (% col)** | **PgR ≥20%**  **N (% col)** | **p** |
| **All** | 9415 (100) | 854 (100) | 2315 (100) |  | 555 (100) | 1721 (100) |  | 1248 (100) | 2722 (100) |  |
| **Age at surgery, years** |  |  |  | <0.001 |  |  | <0.001 |  |  | <0.001 |
| <35 | 309 ( 3.3) | 7 ( 0.8) | 33 ( 1.4) |  | 19 ( 3.4) | 37 ( 2.1) |  | 69 ( 5.5) | 144 ( 5.3) |  |
| 35-50 | 3809 (40.5) | 173 (20.3) | 959 (41.4) |  | 136 (24.5) | 793 (46.1) |  | 396 (31.7) | 1352 (49.7) |  |
| 51-65 | 3637 (38.6) | 499 (58.4) | 924 (39.9) |  | 270 (48.6) | 600 (34.9) |  | 543 (43.5) | 801 (29.4) |  |
| >65 | 1020 (16.6) | 245 (16.8) | 88 (16.8) |  | 181 (17.1) | 81 (22.5) |  | 271 (14.4) | 154 (17.7) |  |
| **Menopausal status** |  |  |  | <0.001 |  |  | <0.001 |  |  | <0.001 |
| Premenopausal | 4278 (45.4) | 201 (23.5) | 1051 (45.4) |  | 150 (27) | 861 (50.0) |  | 477 (38.2) | 1538 (56.5) |  |
| Postmenopausal | 5137 (54.6) | 653 (76.5) | 1264 (54.6) |  | 405 (73) | 860 (50.0) |  | 771 (61.8) | 1184 (43.5) |  |
| **Histology** |  |  |  | <0.001 |  |  | 0.14 |  |  | 0.002 |
| Ductal | 7114 (75.6) | 495 (58.0) | 1543 (66.7) |  | 406 (73.2) | 1306 (75.9) |  | 1094 (87.7) | 2270 (83.4) |  |
| Lobular | 1150 (12.2) | 185 (21.7) | 338 (14.6) |  | 83 (15.0) | 230 (13.4) |  | 80 ( 6.4) | 234 ( 8.6) |  |
| Mixed | 397 ( 4.2) | 38 ( 4.4) | 105 ( 4.5) |  | 35 ( 6.3) | 73 ( 4.2) |  | 30 ( 2.4) | 116 ( 4.3) |  |
| Other | 754 ( 8.0) | 136 (15.9) | 329 (14.2) |  | 31 ( 5.6) | 112 ( 6.5) |  | 44 ( 3.5) | 102 ( 3.7) |  |
| **pT** |  |  |  | 0.13 |  |  | 0.01 |  |  | 0.007 |
| pT1 | 6601 (70.1) | 706 (82.7) | 1918 (82.9) |  | 380 (68.5) | 1279 (74.3) |  | 683 (54.7) | 1635 (60.1) |  |
| pT2 | 2514 (26.7) | 138 (16.2) | 346 (14.9) |  | 152 (27.4) | 398 (23.1) |  | 507 (40.6) | 973 (35.7) |  |
| PT3/4 | 300 ( 3.2) | 10 ( 1.2) | 51 ( 2.2) |  | 23 ( 4.1) | 44 ( 2.6) |  | 58 ( 4.6) | 114 ( 4.2) |  |
| **pN** |  |  |  | 0.09 |  |  | 0.70 |  |  | 0.98 |
| pNx | 309 ( 3.3) | 30 ( 3.5) | 108 ( 4.7) |  | 21 ( 3.8) | 58 ( 3.4) |  | 28 ( 2.2) | 64 ( 2.4) |  |
| pN0 | 5201 (55.2) | 607 (71.1) | 1557 (67.3) |  | 312 (56.2) | 943 (54.8) |  | 561 (45.0) | 1221 (44.9) |  |
| pN+ | 3905 (41.5) | 217 (25.4) | 650 (28.1) |  | 222 (40.0) | 720 (41.8) |  | 659 (52.8) | 1437 (52.8) |  |
| **Grade** |  |  |  | 0.90 |  |  | 0.10 |  |  | <0.001 |
| 1 | 2160 (22.9) | 443 (51.9) | 1223 (52.8) |  | 77 (13.9) | 292 (17.0) |  | 22 ( 1.8) | 103 ( 3.8) |  |
| 2 | 4930 (52.4) | 366 (42.9) | 965 (41.7) |  | 422 (76.0) | 1282 (74.5) |  | 482 (38.6) | 1413 (51.9) |  |
| 3 | 2007 (21.3) | 10 ( 1.2) | 24 ( 1.0) |  | 39 ( 7.0) | 85 ( 4.9) |  | 712 (57.1) | 1137 (41.8) |  |
| Na | 318 ( 3.4) | 35 ( 4.1) | 103 ( 4.4) |  | 17 ( 3.1) | 62 ( 3.6) |  | 32 ( 2.6) | 69 ( 2.5) |  |
| **ER** |  |  |  | 0.001 |  |  | 0.06 |  |  | <0.001 |
| ≥20 | 9228 (98.0) | 839 (98.2) | 2302 (99.4) |  | 543 (97.8) | 1702 (98.9) |  | 1153 (92.4) | 2689 (98.8) |  |
| <20 | 187 ( 2.0) | 15 ( 1.8) | 13 ( 0.6) |  | 12 ( 2.2) | 19 ( 1.1) |  | 95 ( 7.6) | 33 ( 1.2) |  |
| **PVI** |  |  |  | <0.001 |  |  | 0.99 |  |  | 0.71 |
| Absent | 7039 (74.8) | 789 (92.4) | 2035 (87.9) |  | 420 (75.7) | 1302 (75.7) |  | 789 (63.2) | 1704 (62.6) |  |
| Present | 2376 (25.2) | 65 (7.6) | 280 (12.1) |  | 135 (24.3) | 419 (24.3) |  | 459 (36.8) | 1018 (37.4) |  |
| **Surgery** |  |  |  | 0.24 |  |  | 0.04 |  |  | 0.31 |
| Quadrantectomy | 7777 (82.6) | 755 (88.4) | 2010 (86.8) |  | 443 (79.8) | 1438 (83.6) |  | 972 (77.9) | 2159 (79.3) |  |
| Mastectomy | 1638 (17.4) | 99 (11.6) | 305 (13.2) |  | 112 (20.2) | 283 (16.4) |  | 276 (22.1) | 563 (20.7) |  |
| **Radiotherapy** |  |  |  | 0.17 |  |  | 0.88 |  |  | 0.08 |
| No | 1416 (15.0) | 94 (11.0) | 297 (12.8) |  | 86 (15.5) | 262 (15.2) |  | 232 (18.6) | 445 (16.3) |  |
| Yes | 7999 (85.0) | 760 (89.0) | 2018 (87.2) |  | 469 (84.5) | 1459 (84.8) |  | 1016 (81.4) | 2277 (83.7) |  |
| **Chemotherapy** |  |  |  | 0.84 |  |  | 0.004 |  |  | <0.001 |
| No | 6386 (67.8) | 738 (86.4) | 1994 (86.1) |  | 369 (66.5) | 1253 (72.8) |  | 518 (41.5) | 1514 (55.6) |  |
| Yes | 3029 (32.2) | 116 (13.6) | 321 (13.9) |  | 186 (33.5) | 468 (27.2) |  | 730 (58.5) | 1208 (44.4) |  |
| **Competing risk** |  |  |  | 0.94 |  |  | <0.001 |  |  | 0.32 |
| No event | 7149 (75.9) | 718 (84.1) | 1967 (85.0) |  | 394 ( 71) | 1382 (80.3) |  | 827 (66.3) | 1861 (68.4) |  |
| Loco regional relapse | 592 ( 6.3) | 35 ( 4.1) | 88 ( 3.8) |  | 43 ( 7.7) | 93 ( 5.4) |  | 105 ( 8.4) | 228 ( 8.4) |  |
| Distant metastasis | 852 ( 9.0) | 27 ( 3.2) | 68 ( 2.9) |  | 70 (12.6) | 102 ( 5.9) |  | 203 (16.3) | 382 (14.0) |  |
| Other | 822 ( 8.7) | 74 ( 8.7) | 192 ( 8.3) |  | 48 ( 8.6) | 144 ( 8.4) |  | 113 ( 9.1) | 251 ( 9.2) |  |

Abbreviations: ER, estrogen receptor; PgR, progesterone receptor; PVI, peritumoral vascular invasion

**Supplementary table 2**. Multivariate analysis for distant disease free survival

|  | **Model 1** |  | **Model 2** |  |
| --- | --- | --- | --- | --- |
|  | **Hazard Ratio (95% CI)** | **P** | **Hazard Ratio (95% CI)** | **P** |
| **Molecular Subtype** |  |  |  |  |
| Ki-67 <14%, PgR≥20% | Ref |  | Ref |  |
| Ki-67 <14%, PgR<20% | 1.05 (0.67 to 1.65) | 0.82 | 1.04 (0.67 to 1.63) | 0.86 |
| Ki-67 14-19%, PgR≥20% | 1.27 (0.93 to 1.75) | 0.14 | 1.27 (0.93 to 1.75) | 0.13 |
| Ki-67 14-19%, PgR<20% | 2.36 (1.67 to 3.34) | <.0001 | 2.21 (1.57 to 3.13) | <.0001 |
| Ki-67 ≥20%, PgR≥20% | 1.93 (1.45 to 2.58) | <.0001 | 1.88 (1.41 to 2.50) | <.0001 |
| Ki-67 ≥20%, PgR<20% | 1.96 (1.44 to 2.67) | <.0001 | 1.82 (1.33 to 2.48) | 0.0002 |
| **pT** |  |  |  |  |
| pT1 | Ref |  | Ref |  |
| pT2 | 2.14 (1.84 to 2.49) | <.0001 | 2.08 (1.79 to 2.42) | <.0001 |
| pT3/4 | 3.44 (2.68 to 4.41) | <.0001 | 3.38 (2.64 to 4.34) | <.0001 |
| **pN** |  |  |  |  |
| pN0 | Ref |  | Ref |  |
| pN+ | 2.59 (2.18 to 3.08) | <.0001 | 2.12 (1.76 to 2.57) | <.0001 |
| **Grade** |  |  |  |  |
| G1 | Ref |  | Ref |  |
| G2 | 2.20 (1.56 to 3.11) | <.0001 | 2.11 (1.50 to 2.98) | <.0001 |
| G3 | 3.56 (2.45 to 5.17) | <.0001 | 3.28 (2.26 to 4.77) | <.0001 |
| **PVI** |  |  |  |  |
| Absent | Ref |  | Ref |  |
| Present | 1.20 (1.04 to 1.4) | 0.013 | 1.16 (1.01 to 1.35) | 0.04 |
| **Menopausal status** |  |  |  |  |
| Premenopausal | Ref |  | Ref |  |
| Postmenopausal | 1.27 (1.11 to 1.47) | 0.001 | 1.40 (1.21 to 1.62) | <.0001 |
| **Treatment** |  |  |  |  |
| No chemotherapy |  |  | Ref |  |
| Chemotherapy |  |  | 1.56 (1.3 to 1.88) | <.0001 |

Abbreviations: PVI, peritumoral vascular invasion; Ref, reference

Hazards Ratios and 95% confidence intervals (CI) obtained form a multivariable Cox proportional hazards regression model

**Supplementary table 3**. Characteristics of the patients according to the new proposal for molecular subtype definitions based on outcome.

| **Variable** | **Luminal A**  **N (% col)** | **Luminal B**  **N (% col)** | **p** |
| --- | --- | --- | --- |
| **All** | 4890 (100) | 4525 (100) |  |
| **Age at surgery, years** |  |  | <0.001 |
| <35 | 77 ( 1.6) | 232 ( 5.1) |  |
| 35-50 | 1925 (39.4) | 1884 (41.6) |  |
| 51-65 | 2023 (41.4) | 1614 (35.7) |  |
| >65 | 865 (17.7) | 795 (17.6) |  |
| **Menopausal status** |  |  | <0.001 |
| Premenopausal | 2113 (43.2) | 2165 (47.8) |  |
| Postmenopausal | 2777 (56.8) | 2360 (52.2) |  |
| **Histology** |  |  | <0.001 |
| Ductal | 3344 (68.4) | 3770 (83.3) |  |
| Lobular | 753 (15.4) | 397 ( 8.8) |  |
| Mixed | 216 ( 4.4) | 181 ( 4.0) |  |
| Other | 577 (11.8) | 177 ( 3.9) |  |
| **pT** |  |  | <0.001 |
| pT1 | 3903 (79.8) | 2698 (59.6) |  |
| pT2 | 882 (18.0) | 1632 (36.1) |  |
| pT3/4 | 105 ( 2.1) | 195 ( 4.3) |  |
| **pN** |  |  | <0.001 |
| pNx | 196 ( 4.0) | 113 ( 2.5) |  |
| pN0 | 3107 (63.5) | 2094 (46.3) |  |
| pN+ | 1587 (32.5) | 2318 (51.2) |  |
| **Grade** |  |  | <0.001 |
| G1 | 1958 (40.0) | 202 ( 4.5) |  |
| G2 | 2613 (53.4) | 2317 (51.2) |  |
| G3 | 119 ( 2.4) | 1888 (41.7) |  |
| Na | 200 ( 4.1) | 118 ( 2.6) |  |
| **Ki-67** |  |  | <0.001 |
| <14 | 3169 (64.8) | 0 ( 0.0) |  |
| 14-19 | 1721 (35.2) | 555 (12.3) |  |
| >=20 | 0 ( 0.0) | 3970 (87.7) |  |
| **ER** |  |  | <0.001 |
| <20 | 47 ( 1.0) | 140 ( 3.1) |  |
| ≥20 | 4843 (99.0) | 4385 (96.9) |  |
| **PgR** |  |  | <0.001 |
| <20 | 854 (17.5) | 1803 (39.8) |  |
| ≥20 | 4036 (82.5) | 2722 (60.2) |  |
| **PVI** |  |  | <0.001 |
| Absent | 4126 (84.4) | 2913 (64.4) |  |
| Present | 764 (15.6) | 1612 (35.6) |  |
| **Surgery** |  |  | <0.001 |
| Quadrantectomy | 4203 (86.0) | 3574 (79.0) |  |
| Mastectomy | 687 (14.0) | 951 (21.0) |  |
| **Radiotherapy** |  |  | <0.001 |
| No | 653 (13.4) | 763 (16.9) |  |
| Yes | 4237 (86.6) | 3762 (83.1) |  |
| **Chemotherapy** |  |  | <0.001 |
| No | 3985 (81.5) | 2401 (53.1) |  |
| Yes | 905 (18.5) | 2124 (46.9) |  |
| **Competing risk** |  |  | <0.001 |
| No event | 4067 (83.2) | 3082 (68.1) |  |
| Loco regional relapse | 216 ( 4.4) | 376 ( 8.3) |  |
| Distant metastasis | 197 ( 4.0) | 655 (14.5) |  |
| Other | 410 ( 8.4) | 412 ( 9.1) |  |

Abbreviations: ER, estrogen receptor; PgR, progesterone receptor; PVI, peritumoral vascular invasion
